# All-Soft Tissue Meniscus Allograft Transplantation: Indications, Techniques, and Results

**DOI:** 10.1177/26350254221107036

**Published:** 2022-10-11

**Authors:** Bálint Zsidai, Gian Andrea Lucidi, Philipp W. Winkler, Ryan J. Gnandt, Ian D. Engler, Volker Musahl

**Affiliations:** * Department of Orthopaedic Surgery, UPMC Freddie Fu Sports Medicine Center, University of Pittsburgh, Pittsburgh, Pennsylvania, USA; † Department of Orthopaedics, Institute of Clinical Sciences, Sahlgrenska Academy, University of Gothenburg, Gothenburg, Sweden; ‡ Clinica II, IRCCS Istituto Ortopedico Rizzoli, Bologna, Italy; § Department for Orthopaedic Sports Medicine, Klinikum rechts der Isar, Technical University of Munich, Munich, Germany; Investigation performed at Department of Orthopaedic Surgery, University of Pittsburgh Medical Center, Pittsburgh, Pennsylvania, USA

**Keywords:** Knee, MAT, meniscus, allograft, transplantation, all-soft tissue

## Abstract

**Background:**

Meniscus allograft transplantation (MAT) may be indicated for young patients with joint line pain following subtotal or total meniscectomy. Several different approaches for performing MAT have been described in the literature and are influenced by appropriate patient selection, graft-sizing, and soft-tissue fixation techniques. Clinical studies demonstrate favorable results regarding pain relief and knee function in young patients undergoing MAT, making it a viable option for the treatment of postmeniscectomy syndrome.

**Indications:**

Meniscus allograft transplantation is indicated for symptomatic patients following subtotal or total meniscectomy. Selection criteria include patient age below 40 to 45 years, body mass index below 35, chondral changes of grade 2 or less, anatomic or correctable joint alignment, and normal or correctable knee stability.

**Technique Description:**

A standard arthroscopy is performed to confirm the indication for MAT, followed by debridement of the meniscus remnant up to the meniscus-capsular junction. The meniscus horns are prepared using a single No. 5 suture, while 4 to 5 No. 2 sutures are passed through the posterior body. The anterior and posterior root tunnels are drilled, and the meniscus is inserted through a posterior vertical arthrotomy using suture passers transmitted via the bone tunnels. The allograft is manipulated into proper position with a probe and the application of axial traction on the posterior root suture. At this point, sutures attached to the posterior horn are passed to the posterior capsule and 6 to 8 inside-out sutures are used to stabilize and fixate the midbody and anterior portion of the allograft.

**Results:**

Several clinical studies report good outcomes following MAT with a mean survival rate of approximately 70% at 10-year follow-up and 60% at 15 years. Additionally, some evidence is currently available regarding the long-term chondroprotective effect of MAT.

**Description/Conclusion:**

All-soft tissue meniscus allograft transplantation is a feasible approach for substitution of the damaged native meniscus and maintenance of tibiofemoral contact mechanics. Consequently, MAT is an important procedure in the toolkit of knee surgeons providing treatment for young, symptomatic postmeniscectomy patients.

## Graphical abstract

**Graphical Abstract:**
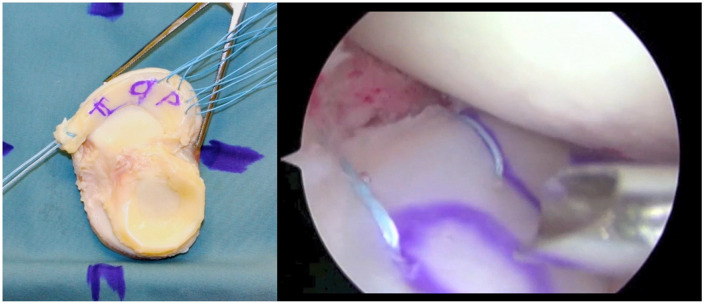
This is a visual representation of the abstract.

## Video Transcript

My name is Volker Musahl, from the University of Pittsburgh. I am presenting on “All-Soft Tissue Meniscus Allograft Transplantation: Indications, Techniques, and Results,” with my coauthors Bálint Zsidai, Gian Andrea Lucidi, Philipp Winkler, Ryan Gnandt, and Ian Engler.

My disclosures are not relevant for this particular presentation.

I’d like to start briefly by giving an overview. This is a beautiful anatomy slide from the late Pau Golano showing the root insertions of the medial and lateral menisci. The selection criteria for meniscus allograft transplantation (MAT) are strict and is essential to consider the appropriate indications. Patient age is usually under 40 years, more often under 30 years. Excessive body weight is a problem; therefore body mass index (BMI) should strictly be below 35. Selection criteria include having had a prior meniscectomy with pain localized at the right of the joint line (as shown in the image) and arthroscopic findings showing ≤ grade 2 chondrosis in the ipsilateral compartment. The contralateral compartment should be free of any significant unaddressed meniscal or chondral injury, alignment of the knee has to be neutral or fixed into neutral and stabilized by a concomitant anterior cruciate ligament (ACL) or posterior cruciate ligament (PCL) reconstruction when required. Advanced osteoarthritis is a very strict contraindication and only minimal Fairbanks changes should be tolerated. Malalignment, as well as instability, need to be corrected. Full range of motion without flexion contracture is a prerequisite. Additional contraindications include previous joint infections and inflammatory arthritis.

Radiographic sizing for MAT should be performed according to Pollard et al^6^ and is a simple and cost-effective method ensuring the use of good lateral and anteroposterior (AP) radiographs for tissue procurement.

Graft preparation begins with a quality check, which should be performed prior to the intubation of the patient, assessing for correct laterality (medial or lateral) and evaluating the actual substance of the meniscus tissue is healthy without any sign of hypoplasia. While graft preparation can be performed using bone or soft tissue only, this video demonstrates the soft tissue only technique.

The case example presents a 26-year-old soccer player with a twisting injury, 2 previous meniscectomies, and persistent medial joint line pain. After nonoperative treatment, the decision was made to proceed with a meniscus transplant. Diagnostic arthroscopy demonstrated intact articular cartilage. The anterior and midbody of the meniscus requires resection, while the posterior aspect is already completely degraded. The remainder of the anterior and midbody can commonly be observed, but it is of no relevance when disrupted. Some of the meniscus can simply be left intact with the transplant placed over it, without debridement all the way to the capsule. However, attaining a bleeding peripheral rim of meniscal tissue is recommended. It is possible, as Freddie Fu has taught, to slightly abrade the edge of the plateau and induce a healing response.^4^

Images of the tunnel preparation display the introduction of an ACL tip aimer into the center of the anterior and posterior root insertions. A 3/32-inch Kirschner (K) wire is drilled, exchanged for a loop suture passer, followed by passing of the sutures. Lateral and medial approaches are created using a mini-incision, 1/3 of which is above and 2/3 below the joint line.

The next image displays the transplant, in this case soft tissue only, which is sharply elevated off the root. No. 5 braided sutures are used in a modified Bunnell fashion for both the anterior and posterior roots. Simple No. 2 braided stay sutures are then passed through the posterior horn (demarcated by lines indicating the anterior and posterior aspects) and will later be passed through the capsule using a free needle.^4^ The root sutures are passed through the anterior and posterior tunnels.

The meniscus is then inserted posterior to the medial collateral ligament (MCL) through a 2.5-cm-long vertical arthrotomy large enough to accommodate the little finger, which roughly matches the size of the meniscus. Once the meniscus is inserted and in the appropriate position (sometimes it is flipped and needs to be rotated with a probe), tension is held on the root insertions, and the inside-out stay sutures are passed through the capsule in a vertical mattress fashion. The capsule is then closed with interrupted No. 0 absorbable braided sutures. Approximately 6 to 8 inside-out vertical mattress sutures are placed, starting at the posterior horn-midbody junction and proceeding anteriorly, until reaching the posterior part of the anterior horn.

Occasionally, the meniscus may be folded, which can be corrected by placing one suture for balance and another underneath the meniscus. Next, the final product is shown, with all sutures in place and the meniscus adequately positioned. With the knee at 60° of flexion, the remaining steps consist of (1) tying the posterior horn vertical mattress sutures superficial to the capsule, (2) tying all of the inside-out sutures, and (3) tying the 2 transosseous root sutures over a bony bridge.

The following video presents an alternative all-inside meniscus transplant technique as described in the *American Journal of Sports Medicine* by Marcacci et al.^2^ In this technique, the meniscus only receives two No. 5 root sutures. The posterior root tunnel is drilled, a suture passing device is applied, and the meniscus is then inserted through a dilated anteromedial portal. Proper positioning of the transplant is achieved using a probe and by placing axial traction on the root suture.

In this technique, the tunnel for the anterior root is drilled at a later stage. After the meniscus transplant has been positioned, all-inside sutures are passed starting at the midportion of the posterior horn. It is important to pass the all-inside sutures deep to the capsule, ensuring the meniscus is held in place. The suture is then tied, anchoring the meniscus to the capsule. This sequence is repeated several times, in this case for a total of 6 sutures. Excessive slack should be removed from sutures without over-tensioning the allograft.

The next images show placement of the second suture at the junction of the posterior horn and midbody, followed by a third suture more anteriorly, a fourth suture posteriorly and a fifth and sixth horizontal mattress suture on the undersurface of the meniscus for balance. The anterior root insertion tunnel is drilled at the location of the anterior horn of the meniscus to avoid any undue over-tensioning of the meniscus allograft. This is crucial for achieving stability of the transplanted meniscus.

The patient is made strictly non-weight-bearing for the first 4 weeks of postoperative rehabilitation, transitioning to progressive weightbearing from 4 to 6 weeks, after which time full weightbearing may be resumed. Range of motion progression is started early but is limited to no more than 90° of flexion during the first 6 weeks. This is followed by closed-chain exercises and muscle strengthening, but there is a complete restriction from running for the first 12 months.

Among the many publications investigating outcomes following MAT,^1,3,5,7,8^ a recent systematic review reports a mean survival rate of approximately 70% at a 10-year follow-up and 60% at 15 years,^5^ which is quite acceptable in cases of salvage procedures.

In summary, meniscus transplantation warrants very strict indications, as many patients undergo secondary procedures (on average 2.5 procedures). Limb alignment should be evaluated preoperatively, with an aggressive stance toward osteotomies to achieve neutral alignment prior to placement of the allograft meniscus. The knee should have ligamentous stability, which may require performing the MAT in conjunction with a primary or (more commonly) revision ACL reconstruction. The intraarticular cartilage should be intact, with at most grade 2 chondral changes. Focal articular cartilage defects can be treated simultaneously or in staged procedures. Allograft extrusion may be a problem seen on magnetic resonance imaging (MRI) and could be countered by medialization of the capsule when necessary.

Good long-term outcomes with return to low-impact sports are possible. However, returning to high-impact athletic activities should be cautioned against. Finally, some quality evidence also supports the chondroprotective effect of meniscus allograft transplantation, thus promoting improved overall long-term knee function.

Thank you very much for your attention!
